# Evaluation of salivary alpha-amylase and cortisol levels in patients undergoing orthodontic mini-implant treatment: a short-term prospective comparative study

**DOI:** 10.1093/ejo/cjaf112

**Published:** 2026-05-26

**Authors:** Ismayil Malikov, Turkan Sezen Erhamza, Himmet Yildirim, Ebru Ilhan Kocak, Osman Caglayan

**Affiliations:** Department of Orthodontics, Faculty of Dentistry, Aksaray University, 68100 Aksaray, Turkey; Department of Orthodontics, Faculty of Dentistry, Dokuz Eylul University, 35390 Izmir, Turkey; Department of Orthodontics, Faculty of Dentistry, Kirikkale University, 71450 Kirikkale, Turkey; Department of Orthodontics, Faculty of Dentistry, Kirikkale University, 71450 Kirikkale, Turkey; Department of Biochemistry, Faculty of Medicine, Kirikkale University, 71450 Kirikkale, Turkey

**Keywords:** orthodontics, mini-implant, dental anxiety, salivary cortisol, salivary alpha-amylase, stress biomarkers

## Abstract

**Background:**

This study aimed to evaluate salivary alpha-amylase (sAA) and salivary cortisol (sC) levels, alongside self-reported dental anxiety, in patients undergoing orthodontic mini-implant (OMI) placement compared with those receiving routine orthodontic treatment (noninvasive follow-up procedures such as archwire or elastic ligature replacement). The primary outcomes were the changes in sC and sAA levels, while the secondary outcome was the dental fear survey (DFS) score. It was hypothesized that OMI placement would elicit stronger physiological and psychological stress responses.

**Methods:**

A short-term prospective comparative study was conducted on 54 participants (27 OMI and 27 control), aged 12–30 years. Dental anxiety was assessed using the DFS. Salivary samples were collected at baseline (T1) and immediately after the procedure (T2) for sAA and sC analysis. Mann–Whitney *U*, Wilcoxon, *t*-tests, and mixed ANOVA were performed (α = 0.05).

**Results:**

sC increased significantly in the OMI group (0.30 ± 0.09 to 0.39 ± 0.13 µg/dl; Δ = +0.09 µg/dl, *P* < .001) but remained stable in controls. The OMI group demonstrated higher DFS scores than controls (63.81 ± 22.59 vs. 40.07 ± 21.28). Female participants reported higher dental fear than males (*P* = .004), and DFS correlated negatively with age (*P* = .014). sAA levels were higher in the OMI group, although neither group nor time effects were significant.

**Conclusions:**

Orthodontic mini-implant placement induces stronger acute stress responses than routine orthodontic treatment, as evidenced by higher dental fear and increased salivary cortisol. These findings highlight the need for enhanced patient management during invasive orthodontic interventions.

## Introduction

Stress is currently characterized as a condition in which the stability of homeostasis is challenged by various internal and external stressors [1]. Exposure to a stressor initiates the activation of two primary biological systems: the sympathetic nervous system (SNS), which enables a rapid response, and the hypothalamic–pituitary–adrenal (HPA) axis, which becomes active with a relative delay [2].

In physiologically healthy individuals, activation of preganglionic sympathetic neurons within the locus coeruleus initiates the sympathetic branch of the autonomic nervous system. This, in turn, stimulates the adrenal medulla to release catecholamines—primarily epinephrine and norepinephrine—into the bloodstream, facilitating the organism’s immediate physiological adaptation to stress [3].

A range of methodologies has been employed to assess SNS activity. Chatterton *et al*. [4] proposed salivary alpha-amylase (sAA) as an indirect indicator of sympathetic activation. Although sAA is primarily a digestive enzyme responsible for starch degradation in the oral cavity and not a direct product of the SNS, numerous studies have demonstrated increased sAA concentrations during both physical and psychological stressors. These elevations have been linked to corresponding changes in NE levels associated with stress responses. Speirs *et al*. [5] reported a rapid elevation in sAA levels shortly after stressor exposure, with a peak occurring ∼10 minutes postonset, followed by a swift decline back to baseline upon cessation of the stressor. Concentrations exhibit a diurnal rise, typically ranging from about 0.43–0.57 μg/dl shortly after awakening to 2.86–8.57 μg/dl in the evening [6].

In emergency situations and cases of acute psychological stress, a physiological stress response is elicited. This response is characterized by the release of epinephrine from the adrenal medulla and increased cortisol levels in blood, saliva, and urine. These alterations are achieved through the activation of the HPA axis. Typically, peak cortisol concentrations are observed 20 to 40 minutes after the initiation of an acute stressor, while recovery to baseline levels generally occurs within 40 to 60 minutes following the stressor's conclusion [7]. Salivary cortisol (sC) in healthy individuals typically peaks at 0.20–1.41 μg/dl in the morning and declines to 0.04–0.41 μg/dl by the afternoon [8].

Numerous studies have confirmed the utility of sAA and sC levels as noninvasive biomarkers for assessing both objective pain intensity and emotional states under stress-inducing conditions [6, 9].

Despite significant technological progress in dentistry, fear of dental treatment persists as a substantial concern and a potentially distressing issue in clinical practice [10]. Various self-report questionnaires have been developed to evaluate dental anxiety, with Kleinknecht’s dental fear survey (DFS) being one of the most widely recognized tools for assessing this condition in adults. The DFS has also been culturally adapted and validated for use in the Turkish population, demonstrating satisfactory psychometric properties [11]. This questionnaire consists of 20 items, each item rated on a scale from 1 to 5 points. Based on the total score, respondents are classified as follows: 0–20 points—no dental fear; 21–40 points—low dental fear; 41–60 points—moderate dental fear; 61–80 points—high dental fear; and scores above 80—extreme dental anxiety [12].

Despite several studies examining stress markers in orthodontic settings, little is known about the acute stress response specifically associated with mini-implant placement, which represents a more invasive intervention compared with routine orthodontic procedures. This study aimed to evaluate and compare dental anxiety levels, along with sC and sAA concentrations, before and after treatment in patients undergoing orthodontic mini-implant placement versus those receiving routine treatment without invasive procedures.

The primary outcomes of this study were the changes in sC and sAA between preprocedure and postprocedure measurements, representing acute biological stress responses. The secondary outcome was the DFS score, reflecting the subjective psychological component of dental anxiety.

The following *a priori* hypotheses were proposed: (i) Orthodontic mini-implant placement would induce greater increases in sC compared with routine orthodontic procedures; (ii) Orthodontic mini-implant placement would induce greater increases in sAA compared with routine orthodontic procedures; (iii) Patients undergoing orthodontic mini-implant placement would report higher DFS scores than those receiving routine treatment.

## Materials and methods

This short-term prospective comparative study was reviewed and approved by the Clinical Research Ethics Committee of Kirikkale University (Approval No: 26/17; date of approval: 21 December 2023). In compliance with the Declaration of Helsinki, informed consent was provided in writing by all participants, and by guardians on behalf of minors, before inclusion in the research. Study samples were prospectively collected from patients attending the Department of Orthodontics at the Faculty of Dentistry, XXX University, between January 2024 and June 2025. All identifying information was excluded to preserve anonymity.

The inclusion criteria for the OMI group were: (i) individuals aged 12–30 years, (ii) those undergoing orthodontic mini-implant placement for the first time, and (iii) subjects in good systemic health with no known history of systemic disease. The exclusion criteria were: (i) individuals taking medications such as antibiotics, antidepressants, or antipsychotics, (ii) those with regular alcohol consumption, (iii) individuals presenting with oral lesions, (iv) those with psychological or psychiatric disorders, (v) participants with a history of salivary gland disease or surgery, and (vi) female subjects who were menstruating, pregnant, or using oral or subcutaneous contraceptives at the time of sampling. This age range (12–30 years) was selected because it represents the adolescent and young adult population in which orthodontic mini-implants are most commonly placed, while also minimizing the influence of age-related variations in stress-responsive endocrine activity that becomes more pronounced in later adulthood.

Using G*Power software (v. 3.0.10, Franz Faul, Universität Kiel, Germany), the power analysis demonstrated that with a significance threshold of 0.05 and an effect size of 0.9, a sample of 54 participants was adequate to reach 90% statistical power. The assumed effect size (Cohen’s *d* = 0.9) was based on previous acute stress studies reporting large changes in both cortisol and alpha-amylase following psychological or procedural stressors [6, 13]. Dental studies have similarly demonstrated meaningful biomarker elevations during orthodontic and routine dental procedures [14, 15]. Although optimistic, this effect size was considered biologically plausible for an invasive intervention such as mini-implant placement. Participants were allocated to two groups: the OMI group (*n* = 27; 7 males and 20 females) and the control group (*n* = 27; 17 males and 10 females). The mean age was 17.3 ± 0.6 years in the OMI group and 18.1 ± 0.7 years in the control group (mean ± SD). All mini-implants were placed by the same orthodontist with more than 5 years of clinical experience to eliminate operator-related variability.

In the control group, routine orthodontic treatment referred to noninvasive follow-up procedures commonly performed during orthodontic visits, such as archwire replacement and elastic ligature renewal. No surgical or anesthetic intervention was performed. Importantly, although the procedure-specific orthodontic interventions themselves were brief, the overall appointment duration was standardized across groups to ensure comparable clinical exposure and anticipatory stress before T2 sampling.

To minimize expectation and communication bias, all patients received the same standardized preprocedural explanation. A single clinician provided a brief, neutral description of the mini-implant procedure, avoiding language that emphasized pain, invasiveness, or risk. The same script was used for all participants, and no additional verbal information was given that could influence anticipatory anxiety.

Dental anxiety was assessed using the Turkish version of the DFS, a validated 20-item questionnaire rated on a 1–5 scale [11]. The instrument was administered at baseline (T1) before any clinical intervention. In this sample, internal consistency of the scale was excellent (Cronbach’s *α* = 0.96).

Because sC and sAA exhibit circadian rhythms and the most diagnostically reliable measurements are obtained in the morning, all participants were scheduled for 09:00 appointments. Participants were instructed to eat and brush their teeth at least 1 hour before the appointment. To minimize the influence of minor daily activities on salivary constituents, they rested for 60 minutes before sampling. Following this rest period, the first saliva samples were collected in the waiting area (T1), and a second set was obtained at the end of the appointment (T2), reflecting the completion of the total chair-time and clinical exposure rather than the duration of the procedure-specific orthodontic intervention alone. Saliva was collected using cortisol-specific tubes with a 16.8-mm diameter (Salivette®, Sarstedt AG & Co., Nümbrecht, Germany). Participants chewed the absorbent swab for 2 minutes. After collection, each specimen was centrifuged at 1000 × g for a duration of 2 minutes in a benchtop centrifuge (Hettich Zentrifugen D-78532, Andreas Hettich GmbH & Co. KG, Tuttlingen, Germany) and stored at −20°C until biochemical analyses. Samples were stored at −20°C for a maximum of 2 weeks before analysis, consistent with stability recommendations for salivary biomarkers. After all collections were completed, sC concentrations were measured with the Elecsys Cortisol II immunoassay on a Cobas e 801 analyzer (Roche Diagnostics, Basel, Switzerland), and sAA activity was measured by an enzymatic colorimetric assay on a Cobas c 703 analyzer (Roche Diagnostics, Basel, Switzerland). sC concentrations were expressed in µg/dl (measurable range: 0.018–63.4 µg/dl; detection limit: 0.018 µg/dl), and sAA activity in U/ml (measurable range: 10–2500 U/ml; detection limit: 10 U/ml). Because Salivette® devices yield a standardized saliva volume during the fixed chewing protocol; individual salivary flow rate was not measured. Accordingly, sAA activity was expressed per milliliter (U/ml) without flow-rate normalization. All biochemical analyses were performed under blinded laboratory conditions. Saliva samples were labeled only with numeric codes that concealed group allocation and participant identity.

IBM SPSS Statistics 27 was used for main analyses; RStudio for data visualization. Descriptive statistics are mean ± standard deviation. Assumptions were checked using Shapiro–Wilk, Levene, and Box’s M tests. Based on these, parametric or nonparametric methods were used. Statistical tests were selected based on the distributional properties of each variable; parametric tests were used when normality and homogeneity assumptions were met, and nonparametric tests were applied when these assumptions were not satisfied. The statistical evaluation was performed using a two-way mixed-design ANOVA together with independent-samples *t*-tests, Mann–Whitney *U* tests, and Wilcoxon signed-rank tests. For the mixed-design ANOVA applied to sAA, the assumptions of normality and homogeneity of variances were satisfied (Shapiro–Wilk and Levene tests, *P* > .05). Although Box’s M test indicated a violation of covariance homogeneity, the ANOVA model was retained because this test is known to be overly sensitive and the present study included balanced groups, conditions under which mixed ANOVA is robust to moderate heterogeneity. Given the balanced group sizes, mixed ANOVA is robust to moderate violations of covariance homogeneity. Continuous variable relationships were assessed per normality assumptions, using Pearson or Spearman correlations. Statistical significance was set at 0.05. Sex-related effects on sC, sAA, and DFS were examined within each group. No significant effects were observed for sC or sAA (*P* > .05); therefore, additional covariate adjustments were not applied.

## Results

All participants provided complete saliva and questionnaire data at both time points; no missing observations occurred for sC, sAA, or DFS scores. The descriptive statistical outcomes for all study variables are displayed in Table 1. Analysis began by examining how gender and age related to other research variables. For gender, females had significantly higher mean DFS scores than males (*U* = 196.000, *Z* = −2.855, *P* = .004), but no significant differences appeared for T1 sC (*U* = 334.000, *Z* = −0.453, *P* = .651), T2 sC (*t*(52) = 0.215, *P* = .831), T1 sAA (*t*(52) = 0.031, *P* = .976), or T2 sAA (*t*(52) = −0.536, *P* = .594). In terms of age, DFS scores correlated negatively and significantly with age (rs = −0.33, *P* = .014), whereas T1 sC (rs = −0.05, *P* = .721), T2 sC (rs = 0.05, *P* = .727), T1 sAA (rs = 0.14, *P* = .310), and T2 sAA (rs = 0.20, *P* = .155) showed no significant correlations with age.

**Table 1:** Baseline comparability and descriptive statistics between the two groups.

Variable	OMI group (*N* = 27)	Control group (*N* = 27)	*P*-value
Gender			.006^a^
Female	20 (74.1%)	10 (37.0%)	
Male	7 (25.9%)	17 (63.0%)	
Age	17.26 ± 3.25	18.07 ± 0.49	.393^b^
DFS score	63.81 ± 22.59	40.07 ± 21.28	<.001^c^
T1 salivary alpha-amylase (sAA; U/ml)	1710.10 ± 672.29	1480.68 ± 770.44	.249^b^
T2 salivary alpha-amylase (sAA; U/ml)	1892.69 ± 798.92	1493.68 ± 765.41	.067^b^
T1 salivary cortisol (sC; µg/dl)	0.30 ± 0.09	0.31 ± 0.17	.672^c^
T2 salivary cortisol (sC; µg/dl)	0.39 ± 0.13	0.31 ± 0.17	.062^b^

Continuous variables are presented as mean ± standard deviation (SD).

^a^χ^2^ test.

^b^Independent-samples *t*-test.

^c^Mann–Whitney *U* test.

sC, salivary cortisol; sAA, salivary alpha-amylase; DFS, dental fear survey; *N*, sample size; T1, baseline sample; T2, postprocedure sample; µg/dl, micrograms per deciliter; U/ml, units per milliliter.

**Figure 1.**
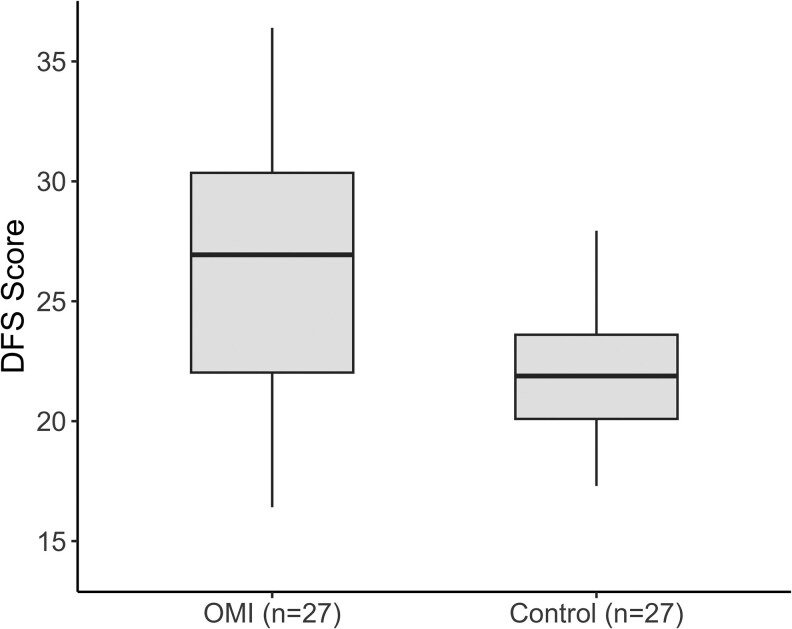
Baseline DFS scores in the OMI and control groups.

After examining demographic variables, we compared DFS scores between the OMI and control groups by using the Mann–Whitney *U* test. Figure 1 illustrates that the OMI group scored significantly higher in DFS than the control group (*U* = 161.000, *Z* = −3.521, *P* < .001).

**Figure 2.**
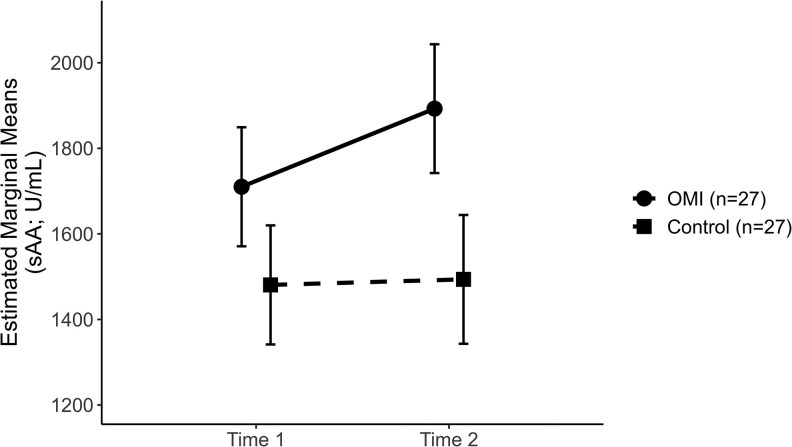
Mean salivary cortisol (sC; µg/dl) levels in the OMI and control groups at T1 and T2. Values represent group means; error bars indicate standard deviations (SD). Sample size: OMI group (n = 27); Control group (n = 27).

A two-way mixed-design ANOVA examined sAA levels. The time-by-group interaction was not significant (*F*(1, 52) = 2.877, *P* = .096). Figure 2 shows that changes in sAA over time did not differ between groups. The main effect of time was not significant (*F*(1, 52) = 3.828, *P* = .056), so mean scores did not differ across measurement points. The main effect of group was also not significant (*F*(1, 52) = 2.497, *P* = .120), indicating no significant difference between groups. The correlation between T1 and T2 sAA was *r* = 0.89, *P* < .001.

**Figure 3.**
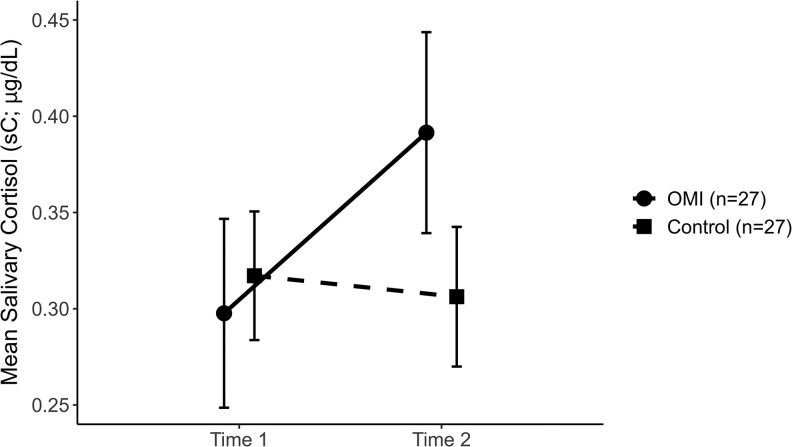
Estimated marginal means of salivary alpha-amylase activity (sAA; U/ml) in the OMI and control groups at T1 and T2. Values represent group means; error bars indicate standard errors (SE). Sample size: OMI group (n = 27); Control group (n = 27).

For sC, the differences between the two measurement points were first calculated for each group (ΔsC). Next, the mean change in sC from T1 to T2 was compared between groups. The Mann–Whitney *U* test found that ΔsC was significantly greater in the OMI group (*U* = 62.000, *Z* = −5.235, *P* < .001). As shown in Fig. 3, sC increased markedly in the OMI group. In contrast, it remained relatively stable in the control group. The Wilcoxon signed-rank test revealed a significant difference between T1 and T2 in the OMI group (*Z* = −3.748, *P* < .001). The difference between the two time points was not significant in the control group (*Z* = −1.048, *P* = .295). When mean differences between groups were examined separately at T1 and T2, no significant differences were observed. At T1, the Mann–Whitney *U* test showed no significant group difference (*U* = 340.000, *Z* = −0.424, *P* = .672). At T2, the independent-samples *t*-test indicated no significant difference between groups (*t*(52) = 1.907, *P* = .062). The correlation between T1 and T2 sC was rs = 0.80, *P* < .001.

## Discussion

Fear reflects a defensive reaction to actual or perceived harm and represents a foundational component of human physiological adaptation [16]. As a multidimensional phenomenon, dental fear integrates physiological responses with behavioral and cognitive processes [17].

Similar to other stress-related conditions, dental fear and anxiety have been linked to alterations in endocrine pathways, particularly those mediated by the adrenal glands. Hans Selye’s [18] 1954 stress theory underscored these adrenal mediators and provided a conceptual basis for later work on dental fear.

Assessment of pain intensity via sAA has been widely explored. Multiple studies suggest that it is particularly useful in populations where self-assessment is not feasible [19-21]. Notably, sAA activity is modulated by multiple factors, primarily physical and psychological stressors [22].

In a cohort of 44 systemically healthy individuals undergoing dental implant therapy (mean age: 62 years), Sabbagh *et al*. [22] assessed the associations of stress with sAA, heart rate, blood pressure, and oxygen saturation. The findings indicated that stress was associated with increased sAA levels and that sAA showed positive correlations with heart rate and oxygen saturation. Extending these results to less invasive procedures, Koh *et al*. [23] examined sAA during venipuncture and reported that, despite venipuncture being less invasive than implant surgery, sAA levels rose during the procedure and remained elevated 15 minutes afterward. Campos *et al*. [24] monitored orthodontic patients over a 21-day period, collecting samples at three stages: pretreatment (days 1–7), bonding (days 8–14), and initial archwire insertion (days 15–21). Their results indicated a progressive increase in salivary sAA across the study period; however, pain intensity did not show a significant correlation with sAA levels. Similarly, Canigur Bavbek *et al*. [25] reported elevated sAA levels during orthodontic treatment, yet this increase was likewise not significantly associated with pain intensity. Similar results were also observed during treatment with self-ligating appliances and clear aligners, with sAA levels increasing particularly within the first 24 hours. These findings highlight the acute impact of orthodontic interventions on stress-related biomarkers [26]. In contrast, research on chronic pain conditions has yielded different findings. Findings from Shirasaki *et al*. [20] and Arai *et al*. [27] indicated that sAA levels increased in relation to pain intensity, implying that this association may differ depending on whether the pain is acute or chronic, as well as on its underlying characteristics. In the present study, prior to the procedure, the OMI group exhibited higher sAA levels in comparison with the control group. sAA levels further increased over time, showing an additional rise at the T2 time point.

Cortisol follows a circadian trajectory characterized by a morning peak at awakening and a gradual decline until evening [28]. Consistent with earlier findings [29], the cortisol response during the most stressful period of dental treatment was referenced to the waking value [29, 30]. Hill *et al*. [31] reported that higher stress and anxiety are associated with elevated sC. In line with these observations, sC assays are widely recognized as accurate and efficient tools for monitoring stress responses. However, elevations can be triggered by both physiological and psychological stimuli, making it difficult to attribute changes to a single factor. This biomarker may also be influenced by intraoral conditions such as dental caries and pulpitis. Supporting this, Gomes *et al*. [32] reported higher sC in children who underwent prophylaxis and presented with caries and/or pulp exposure compared with controls. In contrast, Blomqvist *et al*. [30] observed no significant change in sC among 13-year-old participants who received only radiographic and clinical examinations, a finding that may reflect the absence of any dental intervention. Building on these findings and extending the focus beyond purely physiological triggers, Dušková *et al*. [33] showed that dental-care–related stress elevates cortisol; however, cortisol alone does not quantify the extent to which dental behavior management problems contribute to this rise. A more complete characterization of dental behavior management problems will require incorporating additional biomarkers linked to broader behavioral disorders. Aksoy *et al*. [14] reported that sC increased at the onset of alignment in fixed orthodontic therapy, with the highest values detected during routine appliance insertion and the placement of the first archwire. Consistent with this literature, the present study demonstrated that patients in the OMI group exhibited significantly higher sC levels compared with those in the control group undergoing routine orthodontic procedures, suggesting that the invasive nature of mini-implant placement may amplify the physiological stress response.

Although cortisol most commonly reaches its peak 20–40 minutes after an acute stressor, several physiological studies show that anticipatory stress and sustained task-related stress trigger a progressive rise throughout the procedure itself [34]. In clinical contexts involving orofacial manipulation, patient anxiety increases well before and during the procedure, producing an early HPA-axis activation rather than a delayed isolated peak [35]. Given that each appointment in our setting involved at least 30 minutes of preparation, anticipatory anxiety, and ongoing clinical interaction, rather than prolonged procedure-specific orthodontic intervention, our T2 sampling occurred during a physiologically meaningful window in which cortisol is already elevated.

Unlike cortisol, sAA is driven by very rapid sympathetic–adrenal–medullary activation, resulting in short-lived and highly variable fluctuations [6, 13, 36]. This transient profile, combined with shared anticipatory and procedural stress in both groups, likely masked modest between-group differences. Therefore, the absence of a significant sAA effect reflects the limited discriminatory sensitivity of this biomarker in short orthodontic appointments rather than a lack of sympathetic activation.

Although the OMI group showed statistically higher DFS and sC values than the control group, the absolute stress levels appear to fall within the range of a normal adaptive response rather than extreme distress. For example, studies on tooth extraction or restorative procedures frequently report sC elevations greater than those observed in our sample [37], indicating that OMI-related HPA activation is modest in comparison. Similarly, literature on dental fear shows that DFS scores in typical adult dental populations often fall between 36 and 55, reflecting moderate levels of dental fear rather than severe or phobic anxiety [38]. The DFS means observed in our OMI group remained within this moderate range, and clearly below the high-fear thresholds reported before invasive procedures such as implant surgery [39]. Taken together, these comparisons indicate that mini-implant placement elicits a measurable but clinically moderate stress response—comparable to other routine dental and orthodontic interventions—rather than representing disproportionate or clinically significant psychological distress.

Given the heightened DFS and cortisol responses observed in the OMI group, it is clinically relevant to consider evidence-based strategies commonly used to manage dental anxiety. Established approaches such as structured communication and the “tell–show–do” technique have been shown to improve patient predictability and reduce fear during dental procedures [40]. Behavioral methods including guided breathing and relaxation exercises effectively attenuate autonomic arousal in anxious patients [41]. Cognitive interventions, particularly cognitive reappraisal, can modify threat perception and help patients reinterpret stressful stimuli more adaptively [42]. Adjunctive distraction techniques—such as music, audiovisual aids, or tactile stimulation—further contribute to reducing dental anxiety, especially in individuals with elevated baseline fear [43]. Integrating these well-supported anxiety-management strategies into mini-implant appointments may enhance patient comfort, reduce procedural stress, and improve overall treatment acceptance.

To the best of our knowledge, no previous study has specifically evaluated the acute biological and psychological stress responses associated with orthodontic mini-implant placement in comparison with routine orthodontic procedures.

Some limitations need to be considered. The fact that the investigation was conducted at a single center with a relatively small sample reduces the applicability of the results to broader populations. The unequal gender distribution between groups may have introduced bias. Although the gender distribution differed between groups, additional analyses indicated that sex did not significantly influence sC or sAA levels. This imbalance was acknowledged as a limitation rather than statistically adjusted because of the small sample size. Only short-term changes were assessed, and the absence of long-term follow-up prevented evaluation of recovery or adaptation patterns [44]. Finally, despite standardized morning sampling, individual variability in stress perception, daily habits, and previous dental experiences could still have influenced both biomarker levels and anxiety scores [6, 45]. Another methodological limitation is that the Turkish version of the DFS has been validated only in adults, and no adolescent-specific psychometric validation is available; therefore, the use of the adult form in participants aged 12–17 should be interpreted with caution. Furthermore, behavioral factors known to influence salivary biomarkers—such as sleep quality, recent stress exposure, and caffeine intake—were not systematically monitored. Although all participants were sampled at 9 a.m. following a standardized resting period, these unmeasured variables may have contributed to nonsystematic physiological variability. Additionally, the power analysis was based on a large anticipated effect size (Cohen’s *d* = 0.9), reflecting the substantial biomarker changes reported in acute stress literature; however, such an assumption may be optimistic for clinical orthodontic settings and should therefore be interpreted as a limitation.

Future studies should include larger, multicenter cohorts to improve generalizability and longitudinal follow-up to assess biomarker recovery patterns. Combining salivary markers with additional psychometric tools and autonomic measures could provide a more comprehensive understanding of stress responses. Standardizing behavioral factors and comparing different orthodontic procedures, with or without anxiety-reducing interventions, may further clarify the mechanisms underlying patient anxiety and physiological stress.

## Conclusion

In summary, patients undergoing orthodontic mini-implant placement exhibited significantly higher DFS scores and greater increases in both sC and sAA levels compared with controls, indicating that invasive orthodontic procedures may elicit stronger physiological and psychological stress responses.

## Data Availability

All data generated and/or analyzed for the current study will be made available from the corresponding author upon reasonable request.
